# Cardiometabolic risk burden and associated behaviours in schoolchildren aged 7–10 years from public urban schools in the Federal District, Brazil

**DOI:** 10.1371/journal.pone.0361898

**Published:** 2026-11-02

**Authors:** Shirley Silva Diogo, Muriel Bauerman Gubert, Vivian Siqueira Santos Gonçalves, Ariene Silva do Carmo, Giselle Rhaisa do Amaral e Melo, Giovanna Angela Leonel Oliveira, Natacha Toral

**Affiliations:** 1 Department of Nutrition, Faculty of Health Sciences, University of Brasília, Brasília, Brazil; 2 Graduate Programme in Human Nutrition, Faculty of Health Sciences, University of Brasília, Brasília, Brazil; 3 Department of Collective Health, Graduate Programme in Collective Health, University of Brasília, Brasília, Brazil; 4 Department of Clinical and Social Nutrition, Federal University of Ouro Preto, Ouro Preto, Minas Gerais, Brazil; 5 Department of Nutrition, Centro Universitário IESB, Brasília, Brazil; Tehran University of Medical Sciences, IRAN, ISLAMIC REPUBLIC OF

## Abstract

**Objective:**

To assess cardiometabolic risk burden among schoolchildren aged 7–10 years attending public urban elementary schools in the Federal District, Brazil, and examine its association with health behaviours and socioeconomic characteristics.

**Methods:**

This population-based cross-sectional study included 978 children, with a subsample of 315 undergoing biochemical assessment. Data were collected on dietary intake, eating and sedentary behaviours, physical activity, anthropometry, blood pressure, and biochemical markers. A composite cardiometabolic risk outcome was constructed and assessed only in the biochemical subsample. Analyses accounted for the complex sampling design.

**Results:**

Among children included in the biochemical subsample, 89.9% presented at least one cardiometabolic risk factor and dyslipidaemia was present in 72.0%. In the full sample, overweight was observed in 30.2% of the children. Eating with distractions was associated with higher odds of cardiometabolic risk burden, increasing the likelihood of one to two risk factors (odds ratio = 2.98; 95% confidence interval: 1.62–5.47) and three or more risk factors (odds ratio = 2.52; 95% confidence interval:1.08–5.90). Consuming snacks or treats, compared with a homemade meal, was associated with lower odds of presenting one to two risk factors (odds ratio = 0.32; 95% confidence interval: 0.10–0.99), but no association was observed with three or more risk factors.

**Conclusions:**

Within the non-random biochemical subsample of morning-shift students, a high burden of cardiometabolic risk factors was observed, and eating with distractions was associated with greater cardiometabolic risk burden. Early preventive strategies targeting modifiable behaviours are needed.

## Introduction

Cardiovascular diseases (CVD) represent the leading cause of mortality worldwide, accounting for more than 19 million deaths annually [1]. Although clinical manifestations occur predominantly in adulthood, many underlying risk factors originate earlier in life and may remain undetected during childhood. Despite numerous prevention initiatives targeting adults, the systematic identification and management of cardiovascular risk factors in children remain limited, although this stage of life offers important opportunities for prevention [2].

Evidence indicates that several cardiometabolic risk factors persist from childhood into adulthood, increasing the likelihood of future cardiovascular events [3]. Consequently, childhood represents a key period for the implementation of preventive strategies [4].

In recent decades, the prevalence of cardiometabolic risk factors among children has increased, influenced by biological, behavioural, and social determinants [5]. Biochemical indicators, such as blood glucose, lipid fractions, and C-reactive protein, play a central role in the comprehensive assessment of cardiovascular risk in paediatric populations [6]. At the same time, lifestyle patterns have changed substantially. Evidence increasingly links ultra-processed food consumption, physical inactivity, excessive screen exposure, and eating meals with distractions to adverse cardiometabolic outcomes that are potentially modifiable [2,7,8]. These behaviours are not randomly distributed across populations but are influenced by socioeconomic vulnerability and regional inequalities that affect dietary patterns and opportunities for physical activity [2,9].

Despite advances in the investigation of individual factors, studies assessing the combined burden of multiple cardiometabolic risk factors in children remain limited, particularly in population-based samples with comprehensive biochemical assessment in low- and middle-income countries, such as Brazil [10].

Therefore, this study aimed to characterise the burden of cardiometabolic risk factors among schoolchildren aged 7–10 years attending public urban elementary schools in the Federal District, Brazil, in a non-random biochemical subsample of morning-shift students drawn from a population-based sample with a complex sampling design, integrating biochemical, anthropometric, behavioural, and socioeconomic data.

## Methods

This was a cross-sectional school-based study that followed the STROBE (Strengthening the Reporting of Observational Studies in Epidemiology) guidelines to ensure transparent and standardised reporting of observational research. The study was conducted in accordance with the Declaration of Helsinki and was approved by the Research Ethics Committee of the Faculty of Health Sciences at the University of Brasília (CAAE 17386019.8.0000.0030).

### Study population

The study was conducted between October 3 and December 14, 2022, among children enrolled in the 2nd-4th grades of public urban elementary schools in a central region of Brazil (Federal District).

Schools were selected to ensure representativeness of the target population of schoolchildren enrolled in public schools. Public elementary schools were randomly selected from the 2021 School Census. To capture socioeconomic diversity, schools were stratified according to the Social Vulnerability Index (SVI) of their administrative region [11]: low (SVI < 0.29), medium (SVI 0.30–0.39), and high (SVI ≥ 0.40). The SVI includes indicators of urban infrastructure, human capital, income and employment, and housing conditions.

The sample size was calculated assuming a minimum prevalence of 3%, a relative error of 6%, and a 95% confidence level, considering a non-response rate of up to 25%. This anticipated loss was incorporated into the sampling design based on an average class size of 20 students, estimating participation of approximately 15 students per classroom. Accordingly, three classrooms (clusters) were selected per school, resulting in 60 classrooms across 20 schools, resulting in a minimum sample of 900 schoolchildren.

The final sample included schools across all SVI strata. Within each stratum, schools were selected using probability proportional to size based on enrolment in 2nd - 4th grades. Within the probability proportional to size method, the Pareto method was applied because it leads to lower variability of sampling weights and greater precision of estimates. Since sampling weight variability directly affects estimate variability, the selected sampling method influences the variance of the final estimates. Thus, the selected schools, drawn at random, were invited to participate. All students in the selected classes were invited through a letter sent to parents or legal guardians, accompanied by a written informed consent form. Only children whose parents or legal guardians provided written consent were included in the study.

### Data collection

Data collection followed standardised protocols and included training of the research team and development of a mobile application for the study, with an offline data collection interface and later transfer to Excel spreadsheets.

The procedure was organised in sequential stations to ensure operational efficiency and quality control. Each child participated individually using a research mobile device to complete the questionnaire. The questionnaire included child characteristics (sex and grade), food consumption, eating and sedentary behaviours, and physical activity.

Children’s age and home addresses were obtained from schools to complement sociodemographic information and classify administrative regions according to the SVI.

After the questionnaire, anthropometric measurements (weight and height) and blood pressure were assessed. In a subsample, children attending morning classes returned on a scheduled day for fasting venous blood collection, as described later.

### Study variables

#### Demographic and social vulnerability variables.

The demographic variables included the children’s age, sex, and school grade. To comprehensively characterise the socioeconomic context, the SVI was assessed for both schools and children’s residential areas, using the same typology previously described [11], allowing a better understanding of the social environments in which the children study and live.

#### Food consumption.

Food consumption was assessed using the Food Consumption Questionnaire for Brazilian Schoolchildren – QUACEB (acronym for Questionário Ilustrado de Consumo Alimentar para Crianças Escolares Brasileiras in Portuguese), developed and validated for children aged 7–10 years [12]. The instrument consists of 30 color illustrations depicting foods and dishes commonly consumed by Brazilian children, organised according to food groups and levels of processing defined by the Dietary Guidelines for the Brazilian Population.

Children answered independently, reporting their food intake on the day prior to data collection. Based on the responses, a score for ultra-processed foods was constructed, based on the NOVA food classification and on the simplified instrument described previously [13]. QUACEB items corresponding to ultra-processed foods were organised into 10 subgroups: soft drinks; fruit-based beverages; dairy beverages; packaged salty snacks or savory crackers/cookies; sweet or filled cookies/biscuits or packaged cake; sweets; reconstituted meat products; breads; sauces and spreads; and ready-to-eat meals. Each consumed subgroup of ultra-processed foods received one point, with a maximum score of 10 points. Consumption was classified as adequate (≤4 subgroups) or inadequate (≥5 subgroups).

#### Eating and sedentary behaviours.

Eating and sedentary behaviours were assessed using the Illustrated Questionnaire of Eating and Sedentary Behaviours – QUICAS (acronym for Questionário Ilustrado de Comportamentos Alimentares e Sedentários in Portuguese), a validated illustrated tool for school-aged children [14].

Five eating behaviours were analysed for the previous day: whether the child ate main meals with or without distractions, such as television, tablet, or cellphone; whether they ate accompanied or alone at main meals; whether they kept regular mealtimes for main meals; whether they ate homemade meals (“home-cooked meals”) or processed snacks at main meals; and whether they participated in kitchen-related tasks, such as washing dishes, taking out the trash, cooking, or setting the table. Each behaviour was classified as healthy (eating without distractions, accompanied, at regular time, based on homemade foods, and participating in household tasks) or unhealthy (the opposite behaviours).

Screen use was assessed as a marker of sedentary behaviour, also through the QUICAS. It investigated five types of screens: television, computer, tablet, cellphone, and video games, for the previous day, across three time periods (morning, afternoon, and evening) or “did not watch/use/play.” Excessive screen use was defined as engaging with any type of screen for two or more periods in the previous day [14].

#### Physical activity.

Physical activity was assessed using the Physical Activity Questionnaire for the Previous Day (QUAFDA) [15]. Eleven physical activities were investigated and classified according to intensity (light, moderate, or vigorous). Responses were scored based on estimated energy expenditure, and the total score was used to categorize children as inactive (first tertile) or active (second and third tertiles).

#### Anthropometric Assessment.

Weight and height were measured using calibrated digital scales and portable stadiometers, following the Brazilian Ministry of Health’s protocols [16]. Based on these measures, the Z-score of BMI for age was calculated using World Health Organisation AnthroPlus® software. Overweight was defined as a Z-score > +1 standard deviation (SD).

#### Blood pressure measurement.

Blood pressure was measured according to protocols of the Brazilian Society of Paediatrics [17]. Automatic arm monitors with cuffs sized to each child’s arm circumference were used. Three measurements were taken at five-minute intervals; a fourth was performed if the first two differed by >10 mmHg.

Classification considered age, sex, and height according to established criteria: Normal (<90th percentile), Elevated (≥90th to <95th percentile), and Hypertension (≥95th percentile) [18].

#### Biochemical analyses.

Due to budgetary constraints, biochemical analyses were performed only among children attending morning classes, resulting in a subsample of 315 participants. In addition to financial limitations, this decision was based on the requirement for fasting blood collection, making the morning shift the most appropriate for sample collection. Therefore, the subsample was defined according to the school shift rather than by random selection.

Venous blood was collected at schools during the morning shift after a minimum six-hour fast, including only children who followed the provided instructions. The procedure was performed by trained professionals in appropriate rooms, ensuring comfort and safety.

The following biochemical parameters were analysed: blood glucose, used to detect conditions such as diabetes mellitus; the lipid profile—total cholesterol (TC), high-density lipoprotein cholesterol (HDL-C), low-density lipoprotein cholesterol (LDL-C), and triglycerides (TG)—to identify dyslipidaemias, defined as the presence of an alteration in at least one lipid marker according to adopted guideline cutoffs; and C-reactive protein (CRP), a systemic inflammatory marker.

Table 1 presents laboratory methods, cutoff points, and classifications for each analysis [19].

**Table 1:** Laboratory methods, cut-off points and classification criteria of biochemical parameters (n = 315).

Test	Laboratory Method	Cut-off Point	Classification
Glucose (mg/dL)	Enzymatic – Hexokinase	70–99	Normal
		100–125	Altered
		≥126	Diabetes
Total Cholesterol (mg/dL)	Enzymatic– Esterase/Oxidase	<170	Desirable
HDL-C (mg/dL)	Enzymatic – Colorimetric	>45	Desirable
LDL-C (mg/dL)	Elimination/Catalase Method	<110	Desirable
Triglycerides (mg/dL)	Oxidation/Peroxidation	<75 (7–9 years)	Desirable
		<90 (10–19 years)	
C-reactive protein (mg/L)	Enhanced Latex Immunoturbidimetry	<2	Low Risk
		≥2	High Risk

### Data analysis

Data organisation was performed in Microsoft Excel®, and statistical analyses were conducted in Stata version 17.0 (StataCorp, USA). All analyses incorporated sampling weights, primary sampling units (clusters), and stratification using the svy command to account for the complex survey design.

Sampling weights were calibrated using sex- and age-specific post-strata. The calibrated weight corresponded to the natural design weight multiplied by a calibration factor defined for each sex-and-age post-stratum. Calibration factors were calculated as the ratio between the population total for each post-stratum, obtained from the School Census, and the corresponding estimate generated using the natural design weights, defined as the product of the inverse probabilities of sample inclusion [20,21]. To assess potential selection bias related to the biochemical subsample, sociodemographic, dietary, behavioural, and anthropometric characteristics were compared between children with and without biochemical assessment using the design-adjusted Pearson chi-square test, accounting for the complex survey design (S1 Table). Because biochemical measurements were components of the composite cardiometabolic risk outcome, inferential analyses involving cardiometabolic risk burden were restricted to participants with biochemical data (n = 315). Descriptive analyses included the estimation of the mean age and its corresponding 95% confidence interval (95% CI), as well as weighted proportions with corresponding 95% CIs. Estimates were based on the full sample (n = 978) or the biochemical subsample (n = 315), according to data availability for each indicator.

The outcome variable, cardiometabolic risk factor burden, combined multiple indicators: biochemical alterations (hyperglycaemia, dyslipidaemia, and elevated C-reactive protein), elevated blood pressure, high consumption of ultra-processed foods, physical inactivity, and overweight. For analytical purposes, the variable was categorized into three levels: no risk factors (0), one to two risk factors (1–2), and three or more risk factors (≥3). High consumption of ultra-processed foods and physical inactivity were included as behavioural components of the cardiometabolic risk factor burden, alongside anthropometric, clinical, and biochemical factors. The inclusion of both health behaviours and health factors is consistent with the multidimensional concept of cardiovascular health proposed by the American Heart Association [22]. Other health-related behaviours assessed in the study were not included in the definition of the outcome and were examined as explanatory variables because they represent specific behavioural practices that may be associated with the accumulation of cardiometabolic risk factors.

All explanatory variables were initially included simultaneously in the multinomial regression model, using “no risk factors” as the reference category. A backward elimination procedure was then applied, with variables sequentially removed according to statistical significance [23]. Sex and age were included in all stages of the analysis as adjustment variables and were retained irrespective of statistical significance.

Analyses accounted for the complex sampling design using the svy module, and a 5% significance level was adopted for all analyses.

## Results

A total of 978 children participated in the study, including a subsample of 315 who underwent biochemical assessment. Among the participants, 51.2% were boys, and the mean age was 8.48 years (95% CI: 8.34–8.63) (Table 2).

**Table 2:** Sociodemographic, dietary and behavioural characteristics of schoolchildren in the Federal District, Brazil, 2022 (n = 978).

Variable	%	95% CI
Sex		
Boys	51.2	(47.3–55.1)
Girls	48.8	(44.9–52.7)
Household SVI		
Low	33.7	(19.3-51.9)
Medium	30.8	(16.9–49.3)
High	35.5	(29.6–41.8)
School SVI		
Low	36.2	(20.5–55.5)
Medium	30.3	(15.3–51.2)
High	33.4	(29.6–37.5)
Eating with distractions		
Yes	57.9	(46.3–68.6)
No	42.1	(31.4–53.7)
Eating in company		
Yes	75.6	(68.7–81.4)
No	24.4	(18.6–31.3)
Eating at regular times		
Yes	67.8	(58.1–76.2)
No	32.2	(23.8–41.9)
Type of food at main meals		
Homemade meal (“home-cooked meals”)	88.8	(83.4–92.6)
Processed snack	11.2	(7.4–16.6)
Participation in kitchen activities		
Yes	51.6	(37.8–65.1)
No	48.4	(34.9–62.2)
Daily screen time		
Acceptable (<1 period)	24.9	(18.5–32.7)
Excessive (≥2 periods)	75.1	(67.3–81.5)

CI, confidence interval; SVI, Social Vulnerability Index of the Federal District.

Characteristics of children with and without biochemical assessment were similar for most variables evaluated (S1 Table). A statistically significant difference was observed only for daily screen time (p = 0.021), with excessive screen time being less frequent among children with biochemical assessment (75.4%) than among those without biochemical assessment (81.4%).

Concerning the SVI, children were similarly distributed across household vulnerability levels: 33.7% (95% CI: 19.3–51.9) in low, 30.8% (95% CI: 16.9–49.3) in medium, and 35.5% (95% CI: 29.6–41.8) in high. A comparable pattern was observed for school SVI: 36.2% (95% CI: 20.5–55.5), 30.3% (95% CI: 15.3–51.2), and 33.4% (95% CI: 29.6–37.5), respectively.

With respect to eating behaviours, 57.9% (95% CI: 46.3–68.6) had their meals with distractions such as television or mobile phones. Most children ate with others (75.6%; 95% CI: 68.7–81.4), maintained regular mealtimes (67.8%; 95% CI: 58.1–76.2) and reported consuming homemade meals (“home-cooked meals”) (88.8%; 95% CI: 83.4–92.6). Participation in kitchen-related activities was reported by 51.6% (95% CI: 37.8–65.1). Regarding daily screen time, excessive use (≥2 periods) was observed in 75.1% (95% CI: 67.3–81.5) of the children (Table 2).

Table 3 presents the results for the cardiometabolic risk factors among schoolchildren. Lifestyle-related, anthropometric, and clinical factors were assessed in the full sample (n = 978), whereas biochemical factors and the composite cardiometabolic risk burden were assessed in the biochemical subsample (n = 315). Regarding lifestyle-related factors assessed in the full sample, most children consumed up to four ultra-processed items (84.9%; 95% CI: 81.3–87.9), whereas 15.1% (95% CI: 12.1–18.7) consumed five or more items. In addition, 69.0% (95% CI:47.9–84.3) were physically active.

**Table 3:** Cardiometabolic risk factors among schoolchildren in the Federal District, Brazil, 2022.

Variable	%	95% CI
**Lifestyle-related factors (n = 978)** ^a^		
Ultra-processed food		
Up to 4 items	84.9	(81.3 −87.9)
≥ 5 items	15.1	(12.1 −18.7)
Physical activity		
Inactive	31.0	(15.7–52.0)
Active	69.0	(47.9 −84.3)
**Anthropometric and clinical factors (n = 978)** ^b^		
Blood Pressure		
Normal (< P90)	86.6	(81.5 −90.4)
Elevated BP (≥P90 to <P95)	4.5	(3.1 −6.7)
Hypertension (≥ P95)	8.9	(5.9 −13.2)
Nutritional Status		
Without Overweight	69.8	(66.3 −73.1)
With Overweight	30.2	(26.9-33.7)
**Biochemical cardiometabolic factors (n = 315)** ^c^		
Blood Glucose (mg/dL)		
Hypoglycaemia (<70)	3.1	(0.8–11.2)
Normal (≥ 70 and ≤ 99)	96.6	(89.1–99.0)
Hyperglycaemia (≥ 100)	0.3	(0.0–2.1)
Total Cholesterol (mg/dL)		
Normal (< 170)	67.5	(61.2–73.2)
Elevated (≥ 170)	32.5	(26.8–38.7)
HDL-C (mg/dL)		
Normal (>45)	67.2	(56.9–76.1)
Low (≤ 45)	32.8	(23.9–43.1)
LDL-C (mg/dL)		
Normal (< 110)	76.5	(71.5–80.8)
Elevated (≥ 110)	23.5	(19.2–28.5)
Triglycerides (mg/dL)		
Normal	61.8	(51.2–71.4)
Elevated	38.2	(28.5–48.7)
Dyslipidaemia		
Yes	72.0	(63.0–79.4)
No	28.0	(20.6–37.0)
C-reactive protein (mg/L)		
Low Risk (< 2)	80.6	(74.3–85.6)
High Risk (≥ 2)	19.4	(14.4–25.7)
Burden of Cardiometabolic Risk Factors (n = 315)ᵈ		
0	10.1	(7.7-13.3)
1	29.8	(22.7–37.9)
2	22.8	(16.9–30.2)
3	17.1	(12.6–22.8)
4	11.3	(6.9–17.9)
5	5.9	(3.8–9.0)
6	2.8	(0.8–8.7)
7	0.2	(0.0–1.2)

HDL-C, high-density lipoprotein cholesterol; LDL-C, low-density lipoprotein cholesterol.

^a^Lifestyle-related factors were assessed in the full sample (n = 978).

^b^Anthropometric and clinical factors were assessed in the full sample (n = 978).

^c^Biochemical cardiometabolic factors were assessed only in the subsample of children who underwent biochemical assessment (n = 315).

^d^The composite cardiometabolic risk burden was assessed only in the biochemical subsample (n = 315) and included elevated blood glucose, dyslipidaemia, elevated C-reactive protein, elevated blood pressure (elevated blood pressure: ≥ 90th to <95th percentile; hypertension: ≥ 95th percentile), overweight, high consumption of ultra-processed foods (≥5 items), and physical inactivity.

Within the biochemical subsample, high prevalences of lipid alterations were observed: elevated total cholesterol in 32.5% (95% CI: 26.8–38.7), low HDL-C in 32.8% (95% CI: 23.9–43.1), and elevated LDL-C in 23.5% (95% CI: 19.2–28.5), resulting in dyslipidaemia in 72.0% (95% CI: 63.0–79.4). Elevated triglycerides were identified in 38.2% (95% CI: 28.5–48.7), and elevated C-reactive protein was observed in 19.4% (95% CI: 14.4–25.7).

Also within the biochemical subsample, only 10.1% (95% CI: 7.7–13.3) had no risk factors, 52.6% had one to two risk factors, while 37.3% had three or more risk factors.

Table 4 shows that having meals with distractions, such as television or cellphone use, was the only behaviour statistically associated with the burden of cardiometabolic risk factors. Children who ate with distractions had a lower proportion with no risk factors (4.7% versus 15.0%) and a higher proportion of three or more risk factors (42.2% versus 38.5%) compared to those who ate without distractions (p = 0.0217).

**Table 4:** Distribution of the Burden of Cardiometabolic Risk Factors according to sociodemographic and behavioural variables in the Federal District, Brazil, 2022 (n = 315).

Variable	No risk factors	1–2 risk factors	≥3 risk factors	p-value
**Household social vulnerability index**				0.6674
Low	11.9	48.5	39.6	
Medium	15.9	55.7	28.3	
High	7.3	57.3	35.4	
**School social vulnerability index**				0.5600
Low	13.8	47.0	39.2	
Medium	16.0	55.8	28.2	
High	11.5	51.3	37.2	
**Eating with distractions**				0.0217
Yes	4.7	53.1	42.2	
No	15.0	46.5	38.5	
**Eating in company**				0.6439
Yes	8.0	49.8	42.2	
No	12.2	51.8	36.0	
**Regular mealtimes**				0.3705
Yes	8.3	53.5	38.2	
No	10.4	43.7	45.9	
**Type of main meal**				0.0842
Homemade meal	9.7	51.0	39.3	
Snack or treats	3.5	43.9	52.7	
**Participation in kitchen activities**				0.2097
Yes	9.3	43.2	47.5	
No	8.7	57.5	33.8	
**Daily screen time**				0.8042
Acceptable (≤1 period)	7.8	53.7	38.5	
Excessive (≥2 periods)	9.4	49.0	41.6	

P values were calculated using Pearson’s chi-square test.

Upon conducting the multinomial logistic regression analysis, using children with no cardiometabolic risk factors as the reference category, the behaviour of having meals with distractions showed a statistically significant association with the burden of risk factors. Children exposed to this behaviour had higher odds of presenting one to two risk factors (OR = 2.98; 95% CI: 1.62–5.47; p = 0.002) compared with the reference category. Similarly, higher odds of presenting three or more risk factors were observed (OR = 2.52; 95% CI: 1.08–5.90; p = 0.035) among children who reported having meals with distractions. Regarding the type of main meal, consuming snacks or treats, compared with a homemade meal, was associated with lower odds of presenting one to two risk factors (OR = 0.32; 95% CI: 0.10–0.99; p = 0.048), whereas no statistically significant association was observed for three or more risk factors (OR = 0.71; 95% CI: 0.27–1.84; p = 0.446). The model was adjusted for sex and age.

## Discussion

Among children included in the non-random biochemical subsample of morning-shift students, this study identified a high burden of cardiometabolic risk factors. Most participants (89.9%) presented at least one condition, with the highest prevalence observed for one risk factor (29.8%). These findings indicate a substantial accumulation of cardiometabolic risk factors within the biochemical subsample and highlight the importance of monitoring these conditions from early life stages.

In the full sample, overweight was observed in a relevant proportion of children. Within the biochemical subsample, lipid alterations were frequent, particularly dyslipidaemia and elevated triglycerides. Considered separately according to their respective analytical samples, these findings are consistent with previous evidence from South American children [24]. Differences in study design, population characteristics, and measurement approaches may partly explain differences across studies. The prevalence of lipid abnormalities in the biochemical subsample was higher than previously reported [24]. Blood pressure findings in the full sample were similar to those described in the literature, whereas elevated fasting glucose remained uncommon in the biochemical subsample [24]. In addition, the findings observed in the biochemical subsample are consistent with evidence on metabolic syndrome in children and adolescents from low- and middle-income countries [25].

Biochemical alterations were prominent, especially dyslipidaemia. The prevalence of altered lipid fractions was slightly higher than estimates from national meta-analyses in Brazilian children and adolescents [26]. These comparisons refer to individual components rather than the composite outcome used in this study. Therefore, these comparisons should be interpreted with caution, as differences in outcome definitions may limit direct comparability. Still, they may reflect a continuation of increasing trends in lipid abnormalities in this population [26]. Elevated C-reactive protein was also observed in a relevant proportion of children, consistent with findings from other Brazilian paediatric populations [27].

Sociodemographic characteristics, including sex, age, and the Social Vulnerability Index (SVI), were not associated with cardiometabolic risk burden within the biochemical subsample. This suggests that, within this biochemical subsample, these factors may not directly influence the accumulation of risk. This finding contrasts with some population-based studies [2,5], but is consistent with systematic reviews reporting heterogeneous or null associations [28]. Differences in study design, sample characteristics, and contextual factors may explain these inconsistencies.

The high burden observed within the biochemical subsample may be partly explained by lifestyle changes. These include increased consumption of ultra-processed foods and reduced physical activity among children and adolescents [29,30]. In our sample, a proportion of children reported high consumption of ultra-processed foods and low levels of physical activity. National data also indicate that sedentary behaviour, particularly screen use, is associated with higher consumption of ultra-processed foods among Brazilian schoolchildren [31]. Evidence consistently shows that ultra-processed food consumption is associated with adverse metabolic outcomes, including dyslipidaemia and overweight [32,33].

Although lower levels of ultra-processed food consumption were not classified as a significant risk factor, their presence in children’s diets remains a concern. This is due to their nutritional profile and widespread availability rather than evidence from the present analysis alone. These products are energy-dense, highly palatable, and widely accessible, which may promote excessive intake [34]. Their consumption has been associated with increased risk of non-communicable diseases, including obesity, type 2 diabetes, hypertension, cardiovascular diseases, and mortality, including in middle-income countries [8,32].

Physical inactivity was also included in the composite risk outcome. Evidence indicates that sedentary behaviour and low levels of physical activity are consistent predictors of cardiometabolic risk in childhood [7]. However, daily screen time was not associated with cardiometabolic risk burden within the biochemical subsample. This result should be interpreted cautiously, as screen time may act through indirect pathways, such as dietary behaviours, which were not fully explored in this analysis. This finding differs from previous studies linking screen exposure to unhealthy dietary patterns and cardiometabolic risk [9].

Within the biochemical subsample, eating meals with distractions was associated with cardiometabolic risk burden, with higher odds of both one to two and three or more risk factors. Children exposed to distractions during meals were more likely to have one to two risk factors and three or more risk factors compared with children without this behaviour. Although this association does not imply causality, it suggests that the context of eating may be an important behavioural dimension to consider in future research. This finding highlights the importance of considering not only dietary intake, but also the context in which meals occur.

Within the biochemical subsample, children who consumed snacks or treats had lower odds of presenting one to two cardiometabolic risk factors compared with those who consumed homemade meals. However, this association was not observed for three or more risk factors. This finding should be interpreted with caution, as the association was observed for only one outcome category and the confidence interval was close to the null value. In addition, given the cross-sectional design, this finding should not be interpreted as evidence that consuming snacks or treats has a protective effect.

Some limitations should be considered when interpreting these findings. Due to budgetary constraints, biochemical analyses were performed only in children attending morning classes, resulting in a subsample of 315 participants. In addition, because biochemical testing required fasting, only children attending morning classes were eligible for these analyses. Although the subsample was restricted to morning-shift students, the sampling weights were calibrated using sex- and age-specific post-strata, which may improve representativeness with respect to these characteristics. However, given the non-random selection of the biochemical subsample, its overall representativeness cannot be assured. Comparisons between children with and without biochemical assessment showed similar sociodemographic, dietary, anthropometric, and behavioural characteristics for most variables evaluated, although a difference was observed for daily screen time. As this subsample was defined according to the school shift rather than by random selection, the biochemical findings should be interpreted in light of this sampling strategy. Moreover, the backward elimination procedure was applied to a relatively small biochemical subsample, with the reference category of no risk factors representing only 10.1% of the weighted sample. This data-driven variable-selection procedure may have resulted in instability in variable retention and coefficient estimates and may not fully account for model-selection uncertainty. Therefore, the associations identified in the final model should be interpreted with caution and confirmed in larger samples. In addition, the cross-sectional design precludes causal inferences regarding the observed associations.

## Conclusion

Among children included in the non-random biochemical subsample of morning-shift students, a high burden of cardiometabolic risk factors was observed, and dyslipidaemia was frequent. Within this subsample, eating with distractions was associated with greater cardiometabolic risk burden. These findings contribute to the understanding of early cardiometabolic risk distribution within the biochemical subsample and may inform future research and the development of public health strategies. School-based strategies promoting screen-free meals, reduced consumption of ultra-processed foods, and physical activity may be considered. However, longitudinal studies with larger and randomly selected samples are necessary to better understand temporal relationships and potential causal pathways.

## Supporting information

S1 Table Sociodemographic, dietary and behavioural characteristics of schoolchildren with and without biochemical assessment in the Federal District, Brazil, 2022. (DOCX)
