# Supplementary material for: Cardiometabolic risk burden and associated behaviours in schoolchildren aged 7–10 years from public urban schools in the Federal District, Brazil
Source: PLoS One. 2026 Nov 2;21(11):e0361898. doi: 10.1371/journal.pone.0361898 (PMC13726971; doi:10.1371/journal.pone.0361898)
Supplement: Supplementary file 1 [file pone.0361898.s001.docx]

**S1 Table. Sociodemographic, dietary and behavioural characteristics of schoolchildren with and without biochemical assessment in the Federal District, Brazil, 2022.**

| **Variable** | **Without biochemical assessment (n = 663), % (95% CI)** | **With biochemical assessment (n = 315), % (95% CI)** | **p-value** |
| --- | --- | --- | --- |
| **Sex** |  |  | 0.808 |
| Boys | 51.7 (47.6–55.7) | 50.3 (40.4–60.3) |  |
| Girls | 48.3 (44.3–52.4) | 49.7 (39.8–59.7) |  |
| **Age (years)** |  |  | 0.053 |
| 7 | 30.2 (23.0–38.5) | 15.1 (8.2–26.1) |  |
| 8 | 24.3 (21.0–27.9) | 27.1 (21.4–33.5) |  |
| 9 | 21.3 (16.7–26.8) | 33.9 (26.7–41.8) |  |
| 10 | 24.2 (19.5–29.8) | 24.0 (13.9–38.3) |  |
| **Household SVI** |  |  | 0.734 |
| Low | 33.0 (19.2–50.6) | 35.6 (14.9–63.5) |  |
| Medium | 28.8 (14.9–48.4) | 34.7 (16.2–59.3) |  |
| High | 38.2 (27.9–49.7) | 29.7 (14.2–52.0) |  |
| **School SVI** |  |  | 0.780 |
| Low | 35.4 (18.9–56.2) | 38.1 (15.9–66.8) |  |
| Medium | 28.4 (13.3–50.7) | 34.2 (14.0–62.3) |  |
| High | 36.2 (24.6–49.7) | 27.7 (10.2–56.5) |  |
| **Eating with distractions** |  |  | 0.538 |
| Yes | 55.6 (49.2–61.9) | 58.9 (49.3–67.9) |  |
| No | 44.4 (38.1–50.8) | 41.1 (32.2–50.8) |  |
| **Eating in company** |  |  | 0.411 |
| Yes | 78.6 (75.5–81.4) | 76.2 (69.9–81.5) |  |
| No | 21.4 (18.7–24.5) | 23.8 (18.5–30.1) |  |
| **Eating at regular times** |  |  | 0.583 |
| Yes | 69.5 (65.0–73.8) | 66.8 (57.1–75.3) |  |
| No | 30.5 (26.3–35.0) | 33.2 (24.7–43.0) |  |
| **Type of food at main meals** |  |  | 0.577 |
| Homemade meal (“home-cooked meals”) | 86.8 (82.6–90.2) | 88.3 (83.9–91.6) |  |
| Processed snack | 13.2 (9.8–17.4) | 11.7 (8.4–16.1) |  |
| **Participation in kitchen activities** |  |  | 0.369 |
| Yes | 57.1 (52.5–61.5) | 49.8 (34.1–65.6) |  |
| No | 42.9 (38.5–47.5) | 50.2 (34.5–65.9) |  |
| **Daily screen time** |  |  | 0.021 |
| Acceptable  (<1 period) | 18.6 (15.2–22.5) | 24.6 (19.2–31.0) |  |
| Excessive  (≥2 periods) | 81.4 (77.5–84.8) | 75.4 (69.0–80.8) |  |
| **Ultra-processed food** |  |  | 0.617 |
| Up to 4 items | 84.4 (79.6–88.3) | 85.8 (81.0–89.5) |  |
| ≥5 items | 15.6 (11.8–20.5) | 14.2 (10.5–19.0) |  |
| **Physical activity** |  |  | 0.362 |
| Inactive | 27.5 (13.2–48.8) | 38.1 (15.9–66.8) |  |
| Active | 72.5 (51.2–86.9) | 61.9 (33.2–84.2) |  |
| **Nutritional status** |  |  | 0.155 |
| Without Overweight | 72.1 (66.8–76.8) | 65.2 (57.4–72.3) |  |
| With Overweight | 27.9 (23.2–33.2) | 34.8 (27.7–42.7) |  |

CI, confidence interval; SVI, Social Vulnerability Index of the Federal District.
